# Research on Pedestrian and Cyclist Classification Method Based on Micro-Doppler Effect

**DOI:** 10.3390/s24196398

**Published:** 2024-10-02

**Authors:** Xinyu Chen, Xiao Luo, Zeyu Xie, Defang Zhao, Zhen Zheng, Xiaodong Sun

**Affiliations:** 1 Intelligent Connected Vehicle Development Institute, China FAW Co., Ltd., Changchun 130011, China; chenxinyu8@faw.com.cn (X.C.); xiezeyu@faw.com.cn (Z.X.); zhaodefang@faw.com.cn (D.Z.); zhengzhen3@faw.com.cn (Z.Z.); 2 College of Communication Engineering, Jilin University, Changchun 130000, China; sunxd@jlu.edu.cn

**Keywords:** micro-doppler, fractional polynomial, least squares method, time-frequency analysis, SVM

## Abstract

In the field of autonomous driving, it is important to protect vulnerable road users (VRUs) and ensure the safety of autonomous driving effectively by improving the detection accuracy of VRUs in the driver’s field of vision. However, due to the strong temporal similarity between pedestrians and cyclists, the insensitivity of the traditional least squares method to their differences results in its suboptimal classification performance. In response to this issue, this paper proposes an algorithm for classifying pedestrian and cyclist targets based on the micro-Doppler effect. Firstly, distinct from conventional time-frequency fusion methods, a preprocessing module was developed to solely perform frequency-domain fitting on radar echo data of pedestrians and cyclists in forward motion, with the purpose of generating fitting coefficients for the classification task. Herein, wavelet threshold processing, short-time Fourier transform, and periodogram methods are employed to process radar echo data. Then, for the heightened sensitivity to inter-class differences, a fractional polynomial is introduced into the extraction of micro-Doppler characteristics of VRU targets to enhance extraction precision. Subsequently, the support vector machine technique is embedded for precise feature classification. Finally, subjective comparisons, objective explanations, and ablation experiments demonstrate the superior performance of our algorithm in the field of VRU target classification.

## 1. Introduction

To further reduce the occurrence rate of safety incidents and alleviate drivers’ fatigue, the advanced driver assistance system (ADAS) has become a topic of significant research. The ADAS needs to detect, classify, and track both static and dynamic targets, which proactively alert drivers to potential hazards and protect vulnerable road users (VRUs) [1,2,3]. During the classification process of vulnerable road users (VRUs), the morphological similarity between pedestrians and cyclists frequently leads to misclassification; at the same time, the differences in motion states between these two target groups influence the decision-making framework of ADAS directly. Among the methods for classifying pedestrians and cyclists, the algorithms can be primarily divided into two categories: camera-based target classification and Lidar-based target classification [4,5]. However, the high sensitivity to adverse weather conditions imposes stringent requirements on their application scenarios [6]. Although millimeter-wave radar offers high robustness to environmental conditions, which expands its application scenarios for classifying vulnerable road users, its low capture rate of micro-movement features limits its ability to differentiate between similar targets. Therefore, in this paper, the millimeter-wave radar is identified as a hardware foundation, and its classification algorithm is improved.

Due to the similarity between pedestrians and cyclists, it is difficult for radar Doppler feature recognition models to achieve accurate classification. However, the micro-Doppler effect is an extension of the Doppler effect [7,8,9], which introduces the micro-motion characteristics of objects into the classification process based on the spectral modulation mechanism produced by the movements of the target object. Consequentially, the full utilization of the micro-Doppler effect could improve the efficiency of key feature extraction and elevate the precision of target classification without additional hardware.

Micro-Doppler features, which result from the complex motion states of targets, provide classifiers with detailed structural descriptions, facilitating accurate object recognition and classification [10]. Accordingly, the existing research utilizing micro-Doppler effects to identify pedestrians and cyclists can be roughly divided into two categories: the target classification based on feature extraction [11] and the classification algorithms based on time-frequency differences [12].

In feature extraction approaches, the radar time series are converted into a spectrogram, and neural networks are applied to catch the micro-motion features. For example, Kim, S. et al. transformed radar snapshots of time series into Range-Velocity (RV) images using 2D discrete transforms [13], in which convolutional recurrent units are employed as feature extractors to capture the dynamic characteristics of RV inputs. Pérez, R. et al. proposed a method based on 77 GHz frequency modulated continuous wave (FMCW) single-frame measurements to distinguish pedestrians, cyclists, and cars by extracting radial distance-Doppler-azimuth power spectrum estimates as inputs for convolutional neural networks [14]. Among these two frameworks, a convolutional neural network (CNN) is used for classification [15]. However, the low robustness of CNNs caused a negative impact on classification performance. For enhancing the classification performance of CNNs, Wang, J. et al. combined CNNs and recurrent neural networks (RNNs) to achieve the classification by training and learning the micro-Doppler features in spectrograms of road targets [16]. Unfortunately, the lower computational efficiency of RNNs in dealing with longer sequences makes the RNN-based algorithms’ low applicability in autonomous driving [17,18,19].

In time-frequency differences methods, the micro-Doppler features of the targets are incorporated to facilitate classification. For example, Belgiovane, D. et al. focused on micro-Doppler shifts related to pedestrian and cyclist body parts and bicycle wheels [20], which validated the effectiveness of the micro-Doppler effect in distinguishing road objects. Nevertheless, the periodic micro-Doppler spectral lines produced by rotating wheels lead to misclassification between tricycles and bicycles. Further, Zhang, Y. et al. presented a classification method based on radar echo signal spectrogram envelope features [21], in which the spectrogram containing feature differences was used as the input of the Bayesian network. However, the reliance of Bayesian networks on prior probability information results in a decrease in classification performance. At the same time, Wei, Y. et al. similarly focused on the differences in the Doppler frequency shift envelope of human torso Doppler frequency as micro-Doppler features [22]. The upper and lower envelopes from the filtered grayscale images were obtained by grayscale processing, and the Prewitt operator, and the features were input into a KNN to achieve precise classification. However, the sensitivity to outliers and the deficiencies in processing high-dimensional data of KNNs lead to imbalanced classification weights.

To sum up, neither of the algorithms based on feature extraction and time-frequency differences can achieve accurate classification, which is caused by the insufficient utilization of micro-Doppler features. To address this issue and to accurately classify pedestrians and cyclists, a curve-fitting architecture based on the micro-Doppler effect is designed for this research. Firstly, in radar echo data processing, wavelet thresholding and short-time Fourier transforming are integrated to ameliorate the loss of feature information caused by the high similarity among objects. Subsequently, to enhance the classification capability and robustness of the model, a polynomial fitting module based on scoring was designed in the research, which enhances the model’s sensitivity to the micro-Doppler spectrogram complexity. Finally, to address the issues of the limitations of micro-Doppler feature extraction and the inadequate utilization of micro-motion characteristics, instead of neural networks, SVM is integrated into the algorithm framework to reduce the latency, and the model performance is optimized based on real-world scenario data.

The specific contributions of this paper can be summarized as follows:

- A classification method based on micro-Doppler features is designed to achieve precise classification of pedestrians and cyclists, in which micro-Doppler features are integrated into millimeter-wave radar to achieve accurate and efficient classification;
- The traditional least squares methods are improved by extracting micro-Doppler information with fractional polynomials from radar echo data;
- SVM was applied to radar echo-fitted curves to generate optimal separating hyperplanes and achieve the optimization classification performance of pedestrians and cyclists;
- To comprehensively validate the performance of the proposed framework, in addition to the Car Radar Dataset (CARRADA), a new private dataset (PRIDA) is created to evaluate the algorithm’s performance.

The rest of this paper is organized as follows. In Section 2, we provide the work related to our approach. The materials and methods are described in Section 3. The experimental results and discussion are provided in Section 4. In Section 5, our conclusion and plan are presented.

## 2. Related Work

### Micro-Doppler Echo Signal Model

**Figure 1:**
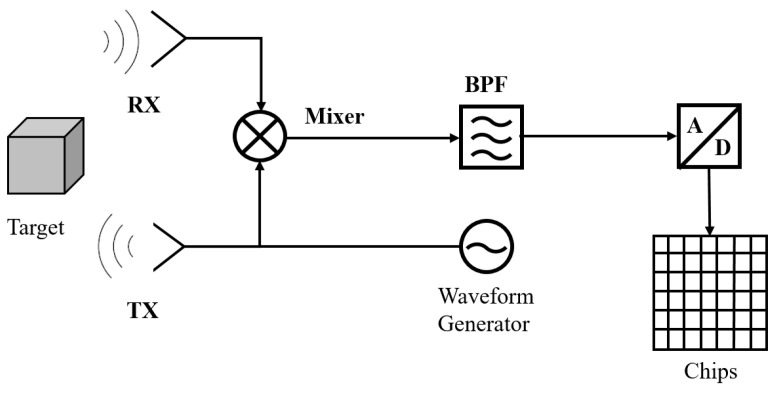
Simplified FMCW radar system block diagram.

In general, FMCW radar systems are widely used in automotive applications because of their superior performance in high-precision distance measurement and motion target detection. In Figure 1, a simplified block diagram of a typical FCWM radar system is shown. Doppler radar systems continuously emit modulated signals and receive reflections from targets, enabling accurate measurements of target distance, azimuth, and relative velocity through frequency shifts.

As for FMCW radar systems, each signal is transmitted by a so-called chirp, which exhibits a linearly varying instantaneous frequency over time. The original transmit signal can be depicted as follows:

(1) $$\begin{array}{l} s(t)=A_{0}\exp(2\pi f_{0}t+2\pi \int_{kT}^{t-kT}f(\tau )d\tau )\\ f(t)=\frac{B}{T}t\\ s_{T}(t)=A_{0}\exp(2\pi (f_{0}t+\frac{B}{2T})t^{2}+\varphi (t))\\ \varphi (t)=-2\pi f_{0}\frac{2r_{p}(t)}{c}\\ f_{d}=\frac{1}{2\pi }\times \frac{d\varphi }{dt}=-\frac{2f_{0}}{c}\times \frac{dr}{dt} \end{array}$$

where the constant $A_{0}$ corresponds to the amplitude of the echo signal, $f_{0}$ and φ(t) are the oscillation frequency and phase noise, t is the instantaneous slant range between radar and target, c is the light speed, $f_{d}$ is the frequency shift, and −dr/dt is the radial velocity relative to the antenna of the target. In addition, regarding a target initially located at a distance $R_{0}$ and moving with velocity v, the received reflected target echo signal with a round-trip delay $t_{d}=2(R_{0}+vt)/c$ is depicted as follows:

(2) $$s_{r}(t)=A_{0}\exp(2\pi (f_{0}(t-t_{d})+\frac{1}{2}\frac{B}{T}{\left(t-t_{d}\right)}^{2}-2kT(t-t_{d})))$$

Equation (2) gives a mathematical overview of the radar echo signal, which shows the Doppler frequency shift is generated by the changes between the emission source and the target. It is directly proportional to the relative motion speed and the emission frequency. Therefore, the Doppler frequency shift information is a typical feature for detecting the motion state of the target. Based on this feature, parameters were estimated to lay the foundation for target imaging, classification, and recognition, such as target structure, size, properties, category, and motion state. The fundamental difference between pedestrians and cyclists lies in their distinct motion postures, where each posture corresponds to different micro-motion features, and each micro-motion feature corresponds to different radar echo signals. As a result, human action recognition can be achieved by extracting micro-Doppler feature information.

## 3. Materials and Methods

### 3.1. Overview

**Figure 2:**
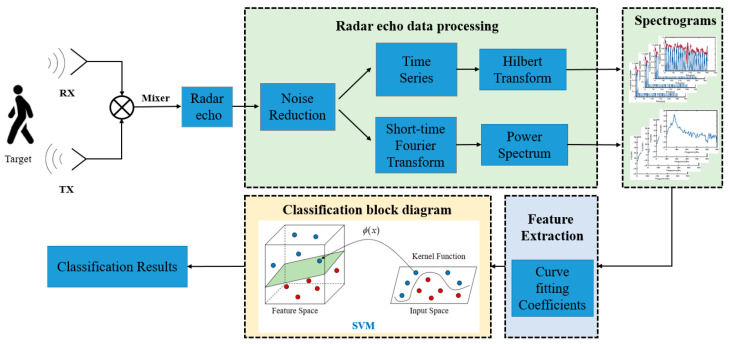
Classification system block diagram for pedestrians and cyclists.

In order to achieve high accuracy and a low model parameter count in micro-Doppler recognition, this paper selects polynomial coefficients of the fractional polynomial as the classification criteria. Fractional polynomial is chosen for its excellent identification performance and generalization ability compared to the traditional least squares methods. In Figure 2, a feature classification architecture for pedestrians and cyclists is shown. This architecture takes advantage of radar echo signal waveforms to estimate both the relative distance and velocity information of targets. The method of curving fitting is utilized to extract micro-Doppler features by converting the obtained data into spectrograms, and these features are fed into a classifier for training to achieve target recognition.

In this study, the recognition model consists of three main components:

1. The radar echo data of pedestrian and cyclist forward movements were de-noised by wavelet threshold processing and transformed from the time domain into the frequency domain using the short-time Fourier transform, and then the power spectrum was estimated by using the periodogram method;
2. The fitting curve coefficients are obtained as the basis for classification by utilizing polynomial fitting of the signal envelope in the time domain and power spectral density in the frequency domain;
3. Feature extracting corresponding to the micro-Doppler features of the targets is learned using the richer curve shapes of fractional polynomials, and classier takes the polynomial coefficients as inputs for a support vector machine model to achieve the recognition of pedestrians and cyclists.

### 3.2. Dataset

Due to the incomplete samples, the public dataset is insufficient to demonstrate the capabilities of the proposed framework fully. To address the issue, PRIDA is constructed to exhibit the performance of the proposed algorithm from multiple perspectives in this paper. The data is sourced from the actual measurements collected from a millimeter-wave radar on open roads, and the primary subjects include pedestrians, cyclists, and tricycle riders, which makes it suitable for contemporary auto-driving perception tasks.

During the data collection process, various challenging driving scenarios, such as low-light conditions, complex targets, and adverse weather, are captured to assess the generalization capability of the detection algorithm.

In PRIDA, 534 continuous sequences are obtained, and over 6000 high-quality objects are labeled. The data acquisition system comprises a high-resolution camera and a 77 GHz millimeter-wave radar, with all sensors calibrated. The camera is directly mounted above the vehicle, while the millimeter-wave radar is installed in the center of the front bumper. Due to the limited horizontal field of view of the camera and radar, data collection is restricted to the area in front of the vehicle. The STA77-5 77 GHz radar provides an approximate horizontal field of view of 80° and a vertical field of view of 8°. Thus, only data within this 80° forward field of view is labeled.

The offline calibration method is used in sensor calibration, and the center point of the vehicle is set as the origin of the multi-sensor relative coordinate system. To synchronize timestamps among the sensors, the GPS messages are employed to ensure the time consistency of the sensing system.

### 3.3. Radar Echo Data Processing

**Figure 3:**
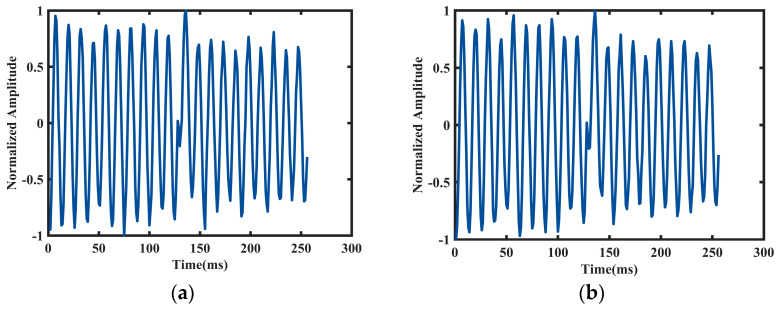
The de-noised radar time-domain echo signal for one cycle is in (**a**) pedestrians and (**b**) cyclists. Relative velocities for a pedestrian walking at a rate of 1.0 m/s and cyclists with a constant speed of 2.0 m/s.

In order to remove the amplitude noise, phase noise, and other interference contained in the signal without altering the primary characteristics of the signal [23], data collection is required to perform noise reduction processing firstly for the human motion model, using the so-called method of wavelet hard threshold de-noising [24,25]. During the experimental process, the data collection is set as a baseline of 3 s, with 100 frames of data collected each time. Examples of relative velocities obtained from the described motion models are shown in Figure 3; both road users move with constant velocities along a linear trajectory toward the radar sensor. While Figure 3a shows the velocities obtained from a male pedestrian model who is walking at a rate of 1.0 m/s, Figure 3b corresponds to the different radial velocities of the cyclists with a constant velocity of v=3.0 m/s. In order to ensure comparability between data across different features and attributes, we employ data normalization procedures to enhance the efficiency and accuracy of data analysis or algorithms.

From the aforementioned two individual radar pulse temporal waveforms, it is difficult for the naked eye to discern the differences between the two target categories. However, the envelope of the radar signal in the time domain corresponds to different micro-Doppler information, which plays an important role in the recognition of pedestrians and cyclists. Additionally, due to the simplicity and ease of implementation, the Hilbert transform method is a commonly used technique in engineering for a continuous-time signal x(t) [26].

(3) $$\begin{array}{l} \overset{-}{x}(t)=x(t)*\frac{1}{\pi t}=\int_{-\infty }^{+\infty }\frac{x(\tau )}{t-\tau }d\tau \\ \overset{-}{X}(f)=X(f)+H(f)\\ F[\frac{1}{\pi t}]=H(f)=-j\mathrm{sgn}(f)=-j\left\{\begin{array}{l} 1\\ -1 \end{array}\right\}\\ \hat{x}(t)=x(t)+j\overset{-}{x}(t) \end{array}$$

Here, $\overset{-}{x}(t)$ and $\overset{-}{X}(t)$ is the Hilbert transform in the time domain and frequency domain, and H(f) is Fourier form of 1/πt.

**Figure 4:**
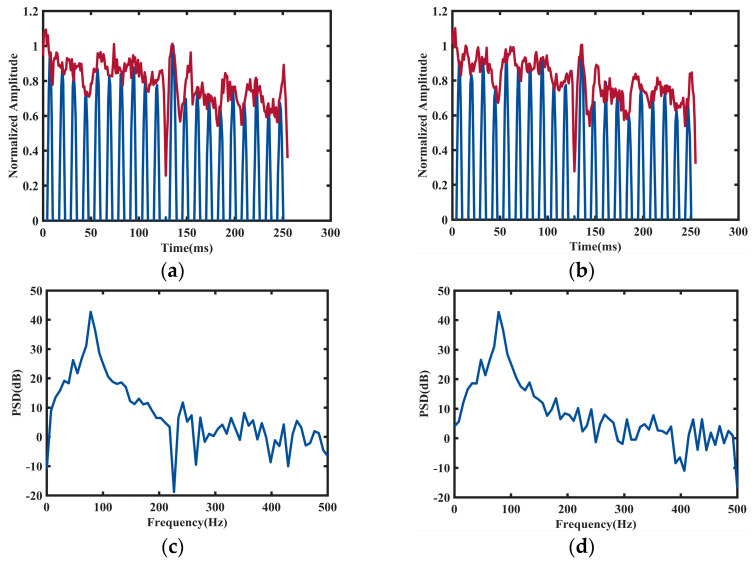
Micro-Doppler signatures of a male pedestrian in (**a**) and a cyclist in (**b**) signal envelope in time-domain from the same scenario. Additionally, (**c**,**d**) show the single-sided power spectrum after STFT.

The amplitude of $\hat{x}(t)$ is the envelope of the signal x(t), which is described in Equation (4). It shows that the amplitude of the Fourier transform of real functions is symmetric. Therefore, this paper only investigates the measurements with a y-axis greater than 0 and extracts the time-domain normalized signal envelope as depicted in Figure 4a,b.

(4) $$A(t)=\sqrt{x^{2}(t)+\overset{-}{x^{2}}(t)}$$

Even though the signal envelope characteristics offer rich micro-Doppler information, it is still difficult to separate the two target categories in most scenarios as their range and velocities are very closed-spaced. In order to mitigate interference among different signal frequencies, micro-Doppler spectrograms are utilized to enhance the performance and reliability of the system. Considering the time-varying and non-stationary characteristics of human motion echoes, a short-time Fourier transform (STFT) method has to be computed over the raw data along the short time dimension [27,28,29]. The core idea is to assume that the selected window function is stationary within each short time interval. The power spectrum can be computed at different moments by moving the preselected window function, effectively converting the non-stationary features of the signal into a series of superimposed short-time stationary signals. The non-stationary signal f(t) undergoes a short-time Fourier transform at time τ as shown in Equation (5):

(5) $$\begin{array}{l} Gf(\omega ,\tau )=\int_{-\infty }^{\infty }f(t)g(t-\tau )e^{-j\omega_{0}t}dt\\ Gf(m,n)=\sum_{n=0}^{N-1}\sum_{m=0}^{M-1}f(k)g(kT-nT)e^{-j\omega_{0}mk} \end{array}$$

where f(k) is the discrete form of f(t), g(w) and g(k) are the windows function, m is the frequency sampling, t is the time sampling, and τ is the time sampling interval. In this work, the STFT of the micro-Doppler signatures for both pedestrians and cyclists are estimated using a Hanning windowing function with a sliding window size of N=216, and then spectral estimation of the signal through a combination of the periodogram method is conducted. By applying the Fourier transform to the observed signal directly, the modulus squared is taken and divided by the data length to obtain the true spectrum estimation of the random signal. Figure 4c,d depicts the single-sided power spectrum after STFT.

In addition, due to the high similarity within the original dimension of the micro-Doppler spectra, it will increase computational complexity for the tracker to estimate the new state vector. As a result, the choice of feature extractor becomes very critical to bringing unique appearance modalities into the tracker and obtaining better discrimination between the targets. The details about different data processing techniques, feature extraction architecture, together with making it compatible with fractional polynomials, and the different optimization functions are addressed in the following Section 3, respectively.

In order to identify inherent patterns in the data through the analysis of curve-fitting related features or parameters, the radar micro-Doppler feature extraction mainly focuses on approximating the functional relationship of discrete point sets by using continuous curves. The least squares method is known as the most widely used method in curve fitting, which estimates the unknown parameters in the observed data model based on the criterion of minimizing the sum of squared errors. However, conventional low-order polynomials can only provide limited shaped curves, and high-order polynomial regression has poor performance and low accuracy. As a result, we adopt the fractional polynomial against the least squares in curve fitting [30,31,32,33]. The power terms in fractional polynomials can be integers or fractions, and since the power terms of the two exhibit an inclusion relationship, integer polynomials can be considered as a subset of fractional polynomials. The advantages of fractional polynomial fitting lie in its concise form and its ability to adapt to various curve shapes of low-order models and approximate asymptotic lines.

### 3.4. Spectrogram Generation

We describe a family of model functions of a single covariate *X*, subject to the restriction X>0, and define a fractional polynomial of degree m to be the function

(6) $$\phi_{m}(X;\xi ,p)=\xi_{0}+\sum_{j=1}^{m}\xi_{j}X^{\left(p_{j}\right)}$$

where *m* is a positive integer, $p=(p_{1},\ldots ,p_{m})$ is a real-valued vector of powers with $p_{1}<\ldots <p_{m}$, and $\xi =(\xi_{0},\xi_{1},\ldots ,\xi_{m})$ are real-valued coefficients. The round bracket notation signifies the Box-Tidwell transformation:

(7) $$X^{\left(p_{j}\right)}=\left\{\begin{array}{ll} X^{p_{j}} & p_{j}\neq 0\\ \ln X & p_{j}=0 \end{array}\right.$$

as using fractional polynomials to fit a set of data, it is necessary to determine the optimal order m and power term values of $p_{j}$. The power term values of $p_{j}$ are usually selected from a pre-set ζ={−2,−1,−0.5,0,1,…,m}. Specifically, the families $\phi_{1}(X;p)$ and $\phi_{2}(X;p)$ have so far found that models with degrees higher than 2 are rarely required in practice. Specially, for m=2 and $p=(p_{1},p_{2})$, definition (6) may be extended to the case of equal powers as $p=(p_{1},p_{1})$

(8) $$\phi_{2}(X;\xi ,p)=\xi_{0}+(\xi_{1}+\xi_{2})X^{\left(p_{1}\right)}$$

a fractional polynomial of degree 1, not 2. However, the limit as $p_{2}$ tends to $p_{1}$ of

(9) $$\begin{array}{l} \xi_{0}+\xi_{1}X^{\left(p_{1}\right)}+\frac{\xi_{2}X^{\left(p_{1}\right)}(X^{\left(p_{2}-p_{1}\right)}-1)}{\left(p_{2}-p_{1}\right)}\\ \xi_{0}+\xi_{1}X^{p_{1}}+\xi_{2}X^{p_{1}}\ln X \end{array}$$

is a three-parameter family of curves.

### 3.5. Feature Extraction

**Figure 5:**
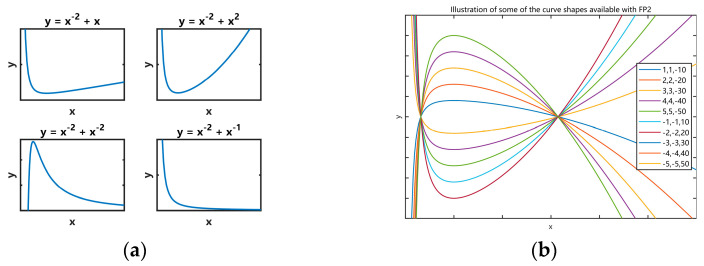
Examples of $\phi_{2}(X;p)$, (**a**) for p=(−2,1),(−2,2),(−2,−2) and (−2,−1), (**b**) a selection of 10 curves with p=(−2,2) using different coefficient vector ξ.

Fractional polynomials with m≤2 offer many potential improvements in fit compared with conventional polynomials, although the family $\phi_{1}(X;p)$ is often useful, but $\phi_{2}(X;p)$ is much richer. It shows that the curves representing $\phi_{2}(X;p)$ can assume four basic shapes, depending on the sign of $\xi_{1}/\xi_{2}$ and on whether $p_{1}$ and $p_{2}$ have the same or different sign. Figure 5a shows examples of the four shapes chosen from the variety available with only one value of $p_{1}$(−2) and four values of $p_{2}$(±1, ±2).

Figure 5b shows a selection of 8 curves available with m=2 and p=(−2,2), using different values of ξ. The ability to generate a variety of curves, some of which have asymptotes or which have both a sharply rising or falling portion and a nearly flat part, is a particularly useful feature of $\phi_{2}(X;p)$.

In summary, the selection of the exponent of the polynomial in the score polynomial requires consideration of the fitted parameter shape through the aforementioned analysis. Different curve shapes represent different fitted parameters, which in turn correspond to different micro-Doppler effects. By extracting micro-Doppler effects, pedestrian and cyclist targets can be distinguished.

In order to thoroughly validate the flexibility of fitting fractional polynomials, we utilize the same power terms to fit the same set of data and introduce the residual sum of squares for comparative analysis. The selection of power terms requires empirical judgment, with the guiding principle being to ensure that the fitting curve closely adheres to the experimental data while minimizing the deviation of the fitted data as much as possible, and it must meet the accuracy requirements for fitting time-frequency domain data. Based on the aforementioned selection criteria, we deliberately selected the most representative schemes of integer polynomial power terms in terms of flexibility and computational speed, namely the 4th and 5th orders, to better contrast the fitting characteristics of integer and fractional polynomials. Within this range, computation is faster, storage space is smaller, and stability is relatively higher. Doing so, the power terms of the integer polynomials selected in the time domain are as follows:

(10) $$\begin{array}{l} a=(a_{0},a_{1},a_{2},a_{3},a_{4})=(0,1,2,3,4)\\ f(t)=a_{0}+a_{1}t+a_{2}t^{2}+a_{3}t^{3}+a_{4}t^{4} \end{array}$$

**Figure 6:**
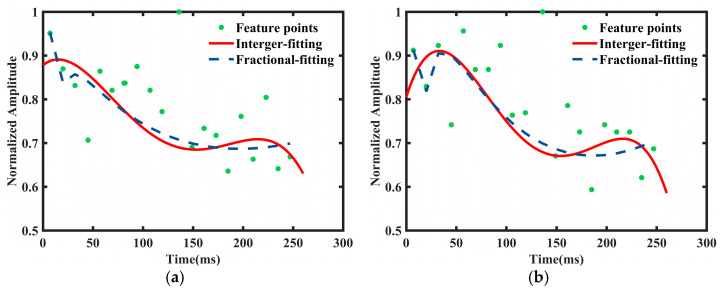
Curve fitting of (**a**) data for pedestrians and (**b**) data for cyclists in the time domain.

In order to keep the same power term with integer, the selection of fractional polynomials is depicted in Equation (11):

(11) $$\begin{array}{l} p=(p_{1},p_{2},p_{3},p_{4},p_{5})=(-2,-1,-0.5,0,2)\\ \phi (x)=\xi_{0}+\frac{\xi_{1}}{x^{2}}+\frac{\xi_{2}}{x}+\frac{\xi_{3}}{\sqrt{x}}+\xi_{4}x^{2} \end{array}$$

using the selected coefficient to extract signatures of pedestrians and cyclists in the time-domain as used for Figure 6a,b.

**Figure 7:**
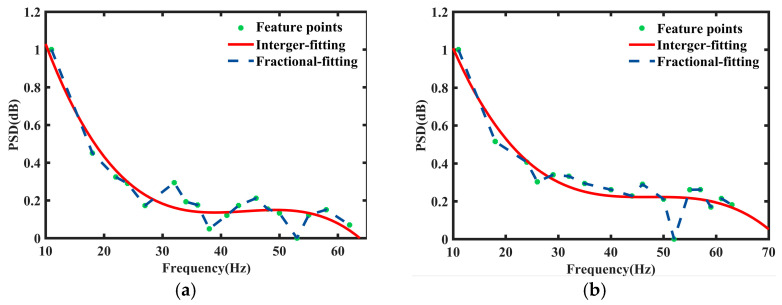
Curve fitting of (**a**) data for pedestrians and (**b**) data for cyclists in the frequency domain.

It can be observed from Figure 5 that fractional polynomials can better fit the data compared to integer polynomials. In order to fully verify the flexibility of fractional polynomials, we use the similarity method for the data in the frequency domain to observe the situation of curve fitting. The comparison between the selected score integer and fractional polynomial in the frequency domain is shown below:

(12) $$\begin{array}{l} b=(b_{1},b_{2},b_{3},b_{4})=(0,1,2,3,4)\\ \mu (t)=b_{0}+b_{1}t+b_{2}t^{2}+b_{3}t^{3}+b_{4}t^{4}\\ k=(k_{1},k_{2},k_{3},k_{4})=(-2,-1,0,1,2)\\ \varphi (x)=\eta_{0}+\eta_{1}(1/x^{2})+\eta_{2}(1/x)+\eta_{3}x+\eta_{4}x^{2} \end{array}$$

using the selected coefficient to extract signatures of pedestrians and cyclists in the frequency domain as used for Figure 7a,b. Coefficients of the integer polynomial fitting curve are listed in Table 1.

Comparing the fitting curves in Figure 6 and Figure 7, it is evident that the curve shapes of the least squares method remain largely consistent when fitting the given data. This implies a lack of sensitivity to local data features, which may result in the loss of critical local information and pose significant challenges in classifying similar targets. Conversely, the fractional polynomial fitting curves closely adhere to the given data, thus showcasing the flexibility of the fitting approach. Additionally, we can compare these two types of polynomials from the perspective of their corresponding fitting parameters. Aiming to minimize the error between data points and the fitted curve, the coefficient of determination of the fitting curve is primarily used to assess the goodness of fit, which can also be utilized to identify trends and patterns in the data, as well as evaluate the effectiveness of different fitting curves.

### 3.6. Classifier

SVM is a class of generalized linear classifiers designed for binary classification through supervised learning. As a binary classification model, its core principle involves defining a linear classifier in the feature space that maximizes the margin between classes. The fundamental approach is to determine a separating hyperplane that not only correctly classifies the training data but also maximizes the geometric margin between the hyperplane and the nearest data points. Prior to broad applicability, capability to address non-linear classification problems, reliance on support vectors, and effectiveness in high-dimensional data processing, SVM is widely employed in tasks including linear and non-linear classification, regression analysis, and anomaly detection.

In this paper, four classifiers, including Naive Bayes (NB), Decision Trees (DT), SVM, and Random Forests (KNN), are used to classify the curve-fitting coefficients of pedestrians and cyclists. The average results of the binary classification are illustrated as follows.

Table 2 presents a comparison between the performance metrics with four classification algorithms evaluated for polynomial coefficients. The results demonstrated that SVM consistently achieves the highest metrics across all evaluation criteria, which demonstrates its exceptional reliability and robustness in classification tasks. Additionally, the SVM model’s lower performance variability further underscores its efficacy, making it a particularly reliable choice for the classification between pedestrians and cyclists within our framework.

## 4. Results and Discussion

### 4.1. Environment Settings and Evaluation Index

To estimate the effectiveness of the research, the private dataset (the data sources from actual radar measurements collected in real road environments) and the Car Radar Dataset (CARRADA) are tested in our experiments. Moreover, an ablation experiment is performed to prove the non-speculative nature of the algorithm in terms of improvement. Four state-of-the-art classification algorithms ([3,17,18,34]) are employed in these experiments to compare with the proposed algorithms

In our experiment, four performance measures, e.g., Accuracy, Precision, Recall, and F1-Score, are calculated using the mean and standard deviation values across multiple cross-validation folds. The formulas used for evaluating the performance of algorithms are detailed as follows:

(13) $$Accuracy=\frac{TP+TN}{TP+TN+FP+FN}$$

(14) $$Recall=\frac{TP}{TP+FN}$$

(15) $$Presion=\frac{TP}{TP+FP}$$

(16) $$F1-Score=2(\frac{Precision\times Recall}{TP+FP})$$

Equations (13)–(16) give a mathematical overview of the performance metrics that is used to assess the efficiency of a classification model. These metrics are computed, including true positive (*TP*), false positive (*FP*), false negative (*FN*), and true negative (*TN*) values. Prior to the evaluation of the classification methods, radial basis function (RBF) kernels were considered from linear, polynomial, and RBF kernels, as RBF particularly demonstrated powerful modeling capabilities in scenarios involving polynomial inseparability or complex nonlinearities.

### 4.2. Results on PRIDA Dataset

#### 4.2.1. Subjective Evaluation of Model Performance

To validate the efficacy of the proposed method, the optimal values of the C=0.1 g=0.01 in the time domain and $C=9.77\times e^{-4}$ g=2.83 in the frequency domain are determined by traversing and searching using the 3-fold cross-validation grid method. Due to space constraints, we randomly selected one out of eight experiments for illustration. The results of optimizing the SVC parameters are shown in the following 3D view.

**Figure 8:**
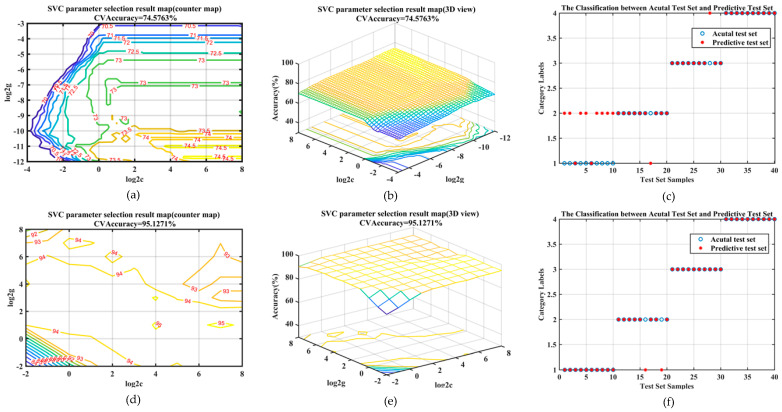
Grid search optimization result map for SVC parameter selection in (**a**,**d**) counter map and (**b**,**e**) 3D view of pedestrian and cyclist in the time domain. Additionally, (**c**,**f**) shows the results of the test dataset among actual samples as well as predictive samples.

As shown in Figure 8a,d, the process of 3-fold cross-validation for parameter optimization on the training set is shown, followed by a grid search to determine the optimal classification accuracy. Figure 8b,e displays the projection of the CV accuracy from Figure 8a,d onto the penalty parameter and RBF kernel function plane. To classify the test set in both time and frequency domains, the C and γ parameters corresponding to the highest average classification accuracy were selected. Consequentially, the classification results on the test set are shown in Figure 8c,f, where all 40 test samples in the time-frequency domain have only 30 correct, while 38 of the 40 groups are in the frequency domain. In order to be subject to certain influences in the process of classification, we propose a random approach during the training of samples to mitigate experimental randomness.

#### 4.2.2. Quantitative Discussion of PRIDA Dataset

In order to explain the advancement and effectiveness of the research comprehensively, this paper randomly selects 40 sets of samples as the test set for each experiment; the average experimental data is listed and analyzed in this section, including indexes Accuracy, Precision, Recall, and F1-Score. The specific data is exhibited in Table 3, and the optimal results are labeled.

As Table 3 shows, the improvement measures designed in our work led to superior performance in all evaluation indexes. Due to the insufficient extraction of micro-Doppler features and the rigid performance of the classifiers, results produced by other comparative experiments are inferior to the effectiveness of the proposed algorithm.

#### 4.2.3. Computational Efficiency Discussion of PRIDA Dataset

In the field of auto-driving applications, in addition to accuracy, the computational efficiency of the algorithm also impacts the superiority of ADAS directly. The algorithms with low latency ensure the real-time responsiveness of the overall system. Therefore, the computational efficiency test of the proposed algorithm is incorporated into the performance evaluation process in this paper.

As shown in Table 4, compared to methods based on deep learning, the proposed demonstrates superior computational efficiency in both training time and runtime, which arises from the complex architecture and more parameters of the deep learning framework. Obviously, the presented algorithm achieves optimal real-time performance while maintaining accuracy.

### 4.3. Results on CARRADA Dataset

Compared to other radar recognition techniques, we undertake a comparative evaluation using the CARRADA radar dataset. This dataset, released by French researchers in 2020, comprised synchronized image and radar data with objects annotated into three categories such as pedestrians, cyclists, and vehicles. Additionally, both indoor and outdoor datasets are used to validate the stability and robustness of the recognition algorithm. In Table 5, the average experimental results are summarized, and the optimal values are marked accordingly.

As Table 5 shows, from the contract experiment, the proposed algorithm consistently performs well in all evaluation indicators employed by our job. Compared to the competing algorithms, our method not only has great performance in accuracy, but also obtains the best F1-score, which directly reflects that the proposed method is competent on the CARRADA dataset. The results demonstrate that our method exhibits superior performance and generalization capabilities in classifying pedestrians and cyclists, outperforming existing techniques.

#### Computational Efficiency Discussion of CARRADA Dataset

In order to show the performance of the algorithm comprehensively, the computational efficiency test based on CARRADA is also added to the experimental process, and the specific experimental results are as follows:

As shown in Table 6, the proposed algorithm still demonstrates the most superior computational efficiency in CARRADA. Therefore, compared to the comparison algorithms, the framework proposed in this paper has the best performance.

### 4.4. Ablation Experiment

In order to verify the effectiveness of the proposed method in this study, several experimental schemes are designed for ablation experiments. Specifically, this includes methods such as noise reduction processing, data normalization, time-frequency analysis, and window function selection. The detailed information is shown in Table 7, with Scheme 5 representing the FP + SVM method used in this paper.

Through these experiments, this paper systematically evaluates the contribution of each improved module to the overall model performance.

The results of each test model are shown based on our dataset in Table 8. Comparing the results of Scheme 1 and Scheme 5, it is shown that replacing the noise reduction processing improves the accuracy by 2.98%. This enhancement underscores the effectiveness of using noise reduction processing.

Comparing Scheme 2 and Scheme 5, it is observed that replacing the data normalization improves the model’s recognition accuracy by 3.26%. Specifically, through noise reduction techniques, the noise within the signal can be attenuated or removed, enabling the enhancement of the signal’s clarity, reliability, and interpretability.

Comparing Scheme 3 and Scheme 5, the introduction of the SFFT improved the accuracy by 2.73%. This improvement is due to the use of the STFT for the precise preservation and characterization of the signal’s localized features. The STFT operates by partitioning the time-domain signal into a sequence of equal-length, quasi-stationary segments using a window function. Each segment is subsequently subjected to the FFT to analyze the temporal evolution of frequency components.

By employing the selection of different window functions, the model can more fully utilize local information from the signal. The Hanning window is characterized by its narrow main lobe width and favorable smoothness properties, which effectively mitigate spectral leakage and enhance the precision of frequency-domain analysis. In addition, the introduction of the Hanning window increases the accuracy by 1.73% when comparing Scheme 4 and Scheme 5, improving the model’s ability to extract detailed features from the spectrogram.

According to the results of each scheme, it is evident that the optimization method proposed in this paper not only enhances the recognition accuracy of pedestrians and cyclists but also enables more accurate differentiation among targets that exhibit similar micro-Doppler features. This capability enhances the model’s applicability in real-world environments.

## 5. Conclusions

Aiming at precise radar classification of VRU targets, this paper proposes a pedestrian and cyclist classification method based on the micro-Doppler effect. Firstly, addressing the classification challenges stemming from the high temporal similarity between pedestrians and cyclists, the conventional time-frequency fusion approach is instead by a new preprocessing model, where only the frequency domain signals of radar echoes are considered in the fitting process of radar echo data. Secondly, to tackle the incomplete classification issue arising from the low inter-class differences between pedestrians and cyclists, fractional polynomials are incorporated into the micro-Doppler feature extraction process of VRUs to enhance classification accuracy. Additionally, SVM is introduced into the feature classification to achieve precise target classification. Finally, the simulation results demonstrate that spectral features achieve higher recognition accuracy for the two target categories, pedestrians and cyclists. At the same time, the fractional polynomial curve fitting strategy not only enhances efficiency but also substantially improves the robustness of the algorithm. Aiming to gradually apply the research findings in practical applications, the author will focus on designing classification methods that are fast in processing and have high accuracy in complex real-world environments in the future.

## Figures and Tables

**Table 1:** Coefficients of the integer polynomial fitting curve.

	$a=(a_{0},a_{1},a_{2},a_{3},a_{4})$	$\xi =(\xi_{0},\xi_{1},\xi_{2},\xi_{3},\xi_{4})$	$a=(a_{0},a_{1},a_{2},a_{3},a_{4})$	$\xi =(\xi_{0},\xi_{1},\xi_{2},\xi_{3},\xi_{4})$
1	[3.18 × 10^−9^, −1.61 × 10^−6^, 2.58 × 10^−4^, −0.02, 0.98]	[−86.57, 37.19, −10.58, −1.27 × 10^−5^, 1.32]	[−1.44 × 10^−9^, 5.67 × 10^−7^, −6.18 × 10^−5^, 0.04, 0.21]	[−510.36, 141.92, −34.93, −4.63 × 10^−6^, 2.58]
2	[2.69 × 10^−9^, −1.38 × 10^−6^, 2.26 × 10^−4^, −0.02, 1.11]	[−22.02, 1.49, 1.39, −8.38 × 10^−6^, 0.60]	[−1.19 × 10^−9^, 4.85 × 10^−7^, −6.02 × 10^−5^, 0.05, 0.25]	[−618.90, 172.87, −44.97, −1.15 × 10^−5^, 3.41]
3	[3.72 × 10^−9^, −1.92 × 10^−6^, 3.16 × 10^−4^, −0.02, 0.92]	[−86.75, 28.90, −6.73, −1.07 × 10^−5^, 1.19]	[−3.86 × 10^−10^, −4.04 × 10^−7^, 1.01 × 10^−4^, 0.04, 0.24]	[−465.50, 116.15, −28.58, −7.11 × 10^−6^, 2.36]
4	[2.63 × 10^−9^, −1.36 × 10^−6^, 2.15 × 10^−4^, −0.01, 0.90]	[−136.87, 46.78, −11.86, −1.28 × 10^−5^, 1.46]	[−1.47 × 10^−9^, 6.41 × 10^−7^, −9.24 × 10^−5^, 0.04, 0.11]	[−583.98, 175.31, −44.97, −1.02 × 10^−5^, 3.40]
5	[3.30 × 10^−9^, −1.52 × 10^−6^, 2.17 × 10^−4^, −0.01, 0.99]	[−9.27, 16.31, −6.39, −1.38 × 10^−5^, 1.28]	[−2.38 × 10^−9^, 8.93 × 10^−7^, −8.99 × 10^−5^, 0.04, 0.22]	[−512.29, 134.41, −33.60, −6.68 × 10^−6^, 2.58]
	$b=(b_{0},b_{1},b_{2},b_{3},b_{4})$	$\eta =(\eta_{0},\eta_{1},\eta_{2},\eta_{3},\eta_{4})$	$b=(b_{0},b_{1},b_{2},b_{3},b_{4})$	$\eta =(\eta_{0},\eta_{1},\eta_{2},\eta_{3},\eta_{4})$
6	[7.49 × 10^−7^,−1.3 × 10^−4^, 8.72 × 10^−3^, −0.25, 2.86]	[−93.51, 40.14, 0.06, −5.31 × 10^−4^, −2.52]	[−2.76 × 10^−9^, 1.4 × 10^−6^, −2.21 × 10^−4^, 0.01, −0.84]	[224.57, −24.22, −0.03, 2.59 × 10^−4^, 1.70]
7	[7.36 × 10^−7^, −1.4 × 10^−4^, 9.37 × 10^−3^, −0.16, 3.02]	[−168.17, 53.51, 0.08, −5.8 × 10^−4^, −3.23]	[−2.24 × 10^−9^, 1.16 × 10^−6^, −1.92 × 10^−4^, 0.01, −0.84]	[149.18, −3.62, −2.13, 1.96 × 10^−5^, 0.11]
8	[9.12 × 10^−7^, −1.6 × 10^−4^, 0.01, −0.30, 3.20]	[−209.84, 52.67, 0.05, −0.71 × 10^−4^, −2.60]	[−1.26 × 10^−9^, 6.71 × 10^−6^, −1.18 × 10^−4^, 0.01, −0.89]	[37.88, 16.17, 0.03, 2.41 × 10^−4^, 1.07]
9	[5.44 × 10^−5^, −1.0 × 10^−4^, 7.28 × 10^−3^,−0.23, 2.73]	[−190.11, 53.28, 0.07, −5.37 × 10^−4^, −2.94]	[−1.53 × 10^−9^, 7.62 × 10^−6^, −1.17 × 10^−4^, 0.01, −0.90]	[177.05, −11.26, −0.02, 1.78 × 10^−4^, 0.78]
10	[8.72 × 10^−5^, −1.6 × 10^−4^, 0.01, −0.28, 3.08]	[−88.88, 36.54, 14, 0.06, −4.71 × 10^−4^, −2.16]	[−1.91 × 10^−9^, 8.76 × 10^−6^, −1.32 × 10^−4^, 0.01, −0.88]	[101.96, 1.80, 0.05, −4.73 × 10^−5^, −0.05]

**Table 2:** Performance metrics of classification by using four classifiers.

Classification Algorithms	Accuracy	Precision	Recall	F1-Score
Fitting Coefficients-NB	0.9127	0.9153	0.9132	0.9264
Fitting Coefficients-DT	0.9174	0.9203	0.9195	0.9232
Fitting Coefficients-SVM	0.9279	0.9379	0.9318	0.9473
Fitting Coefficients-KNN	0.9207	0.9267	0.9219	0.9338

**Table 3:** Average values on PRIDA of comparison and the proposed framework.

Work	Input Features	Model Details	Accuracy	Precision	Recall	F1-Score
[34]	Micro-Doppler Spectrogram	ResNet + Mish	0.8913	0.9187	0.9123	0.9214
[17]	Micro-Doppler Spectrogram	CNN + RNN	0.8827	0.8931	0.8909	0.9002
[18]	Micro-Doppler Spectrogram	DCNN	0.9181	0.9273	0.9198	0.9277
[3]	Micro-Doppler Spectrogram	Bayesian + KNN	0.9452	0.9538	0.9477	0.9592
our	Micro-Doppler Spectrogram	FP + SVM	0.9734	0.9846	0.9688	0.9875

**Table 4:** Computational time on PRIDA.

Work	Model Details	Model Training Time (min)	Total Running Time (min)
[34]	ResNet + Mish	476.27	476.27
[17]	CNN + RNN	634.15	634.15
[18]	DCNN	576.86	576.86
[3]	Bayesian + KNN	398.77	398.77
our	FP + SVM	8.39	8.39

**Table 5:** Average values on CARRADA of comparison and the proposed framework.

Work	Input Features	Model Details	Accuracy	Precision	Recall	F1-Score
[34]	Micro-Doppler Spectrogram	ResNet + Mish	0.9165	0.9231	0.9187	0.9282
[17]	Micro-Doppler Spectrogram	CNN + RNN	0.8979	0.9063	0.9012	0.9175
[18]	Micro-Doppler Spectrogram	DCNN	0.9064	0.9132	0.9118	0.9244
[3]	Micro-Doppler Spectrogram	Bayesian + KNN	0.9218	0.9347	0.9285	0.9479
our	Micro-Doppler Spectrogram	FP + SVM	0.9673	0.9703	0.9689	0.9647

**Table 6:** Computational time on CARRADA.

Work	Model Details	Model Training Time (min)	Total Running Time (min)
[34]	ResNet + Mish	521.89	521.89
[17]	CNN + RNN	698.26	698.26
[18]	DCNN	593.72	593.72
[3]	Bayesian + KNN	407.98	407.98
our	FP + SVM	12.57	12.57

**Table 7:** Experimental design.

Scheme Number	Noise Reduction Processing		Data Normalization		Time-frequency Analysis		Window Function	
	Not Use	Use	Not Use	Use	STFT	FFT	Hanning	Rectangular
1	✓			✓	✓		✓	
2		✓		✓	✓			✓
3		✓	✓		✓		✓	
4		✓		✓		✓		
5		✓		✓	✓		✓	

**Table 8:** Results of each scheme for PRIDA.

Scheme Number	Accuracy	Precision	Recall	F1-Score
1	0.9436	0.9542	0.9483	0.9566
2	0.9406	0.9507	0.9332	0.9518
3	0.9461	0.9484	0.9471	0.9508
4	0.9561	0.9682	0.9437	0.9698
5	0.9734	0.9846	0.9688	0.9875

## Data Availability

Part of data are contained within the article.
